# Rationing Antiretroviral Therapy for HIV/AIDS in Africa: Choices and Consequences

**DOI:** 10.1371/journal.pmed.0020303

**Published:** 2005-09-20

**Authors:** Sydney Rosen, Ian Sanne, Alizanne Collier, Jonathon L Simon

## Abstract

The question facing African governments and societies, say Rosen and colleagues, is not whether to ration such therapy, but how to do so in a way that maximizes social welfare.

In the past three years, expanding access to antiretroviral therapy (ART) for HIV/AIDS has become a global objective and a national priority for many countries in sub-Saharan Africa. Large-scale treatment programs have been launched in countries spanning the continent from Lesotho to Ghana, paid for by domestic funds mobilized by African governments and by international donor contributions.

While these funds, which reach into the billions of dollars, will pay for ART for many thousands of HIV-positive Africans, there is almost no chance that African countries will have the human, infrastructural, or financial resources to treat everyone who is in need. National plans for treatment rollout typically call for a specific number of patients to initiate therapy within the first one or two years of the program. Though the target patient numbers are extremely ambitious—often requiring a 10-fold expansion of services over a two-year period—they still represent a minority of those who are eligible for antiretrovirals on even the most conservative medical grounds. Table 1 indicates the demand for and supply of ART in several African countries and globally, based on starting ART at a CD4 count of 200 cells/μl or an AIDS-defining illness.

**Table 1:**
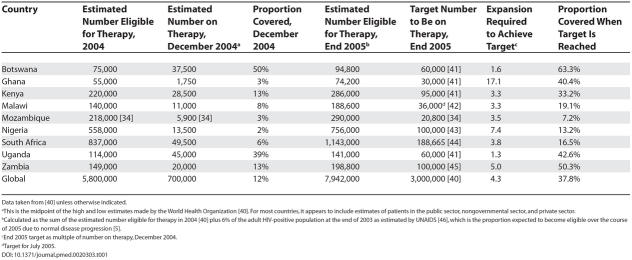
Targets for Treatment Coverage in Selected African Countries and Globally

Data taken from [40] unless otherwise indicated.

^a^ This is the midpoint of the high and low estimates made by the World Health Organization [40]. For most countries, it appears to include estimates of patients in the public sector, nongovernmental sector, and private sector.

^b^ Calculated as the sum of the estimated number eligible for therapy in 2004 [40] plus 6% of the adult HIV-positive population at the end of 2003 as estimated by UNAIDS [46], which is the proportion expected to become eligible over the course of 2005 due to normal disease progression [5].

^c^ End 2005 target as multiple of number on therapy, December 2004.

^d^ Target for July 2005.

The message of Table 1 is clear: rationing of ART is already occurring and will persist for many years to come. The question facing African governments and societies is not whether to ration ART, but how to do so in a way that maximizes social welfare, now and in the future.

Inevitably, the social and economic consequences of rationing a scarce and valuable resource—treatment for a life-threatening illness—will vary widely depending on the rationing system chosen. In a previous article [1], we argued that the chances of achieving a socially desirable outcome from the global intervention now being launched will be higher if an open public-policy debate is conducted and policies are selected that make transparent the trade-offs inherent in any rationing system. We also identified a number of possible rationing systems and proposed several criteria that could be used to select among them. In this paper, we examine these issues in more detail and use an expanded set of criteria to evaluate several rationing systems that already exist in sub-Saharan Africa.

## Systems for Rationing

In economic terms, any policy or practice that restricts consumption of a good is a rationing system [2]. A rationing system restricts demand for a scarce resource so that it matches supply [3]. In the marketplace, price is the basis for rationing: those who can and are willing to pay the market price obtain the resource, while those who cannot or will not pay go without. Nonmarket goods, such as access to free or subsidized medical care, are rationed in a variety of other ways [4]. As used by economists, rationing is a morally neutral concept. It does not imply an intent to deprive some people of a good, but rather describes the allocation of a resource of which there is not enough to go around. Non-price rationing of health care has a long history and is widespread and accepted in many parts of the world, reflecting the widely held view that access to health care should be based on some notion of need, and not determined solely by ability to pay [4]. At the same time, non-price rationing is inherently political. It can be, and often is, used to channel resources toward or away from particular groups for reasons unrelated to their absolute or relative need for the resource. [Fig pmed-0020303-g001]

**Figure pmed-0020303-g001:**
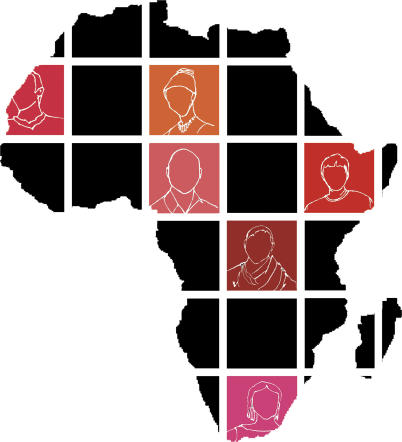
Different approaches to rationing HIV drugs will have different social and economic consequences for African populations (Illustration: Margaret Shear)

In this paper, we define an ART rationing system as any allocation of public resources that prioritizes access to HIV/AIDS treatment on the basis of any geographic, social, economic, cultural, or other nonmedical factor. This is important, as virtually all programs will set a medical threshold for access to treatment, in most cases having a CD4 count lower than 200 cells/μl or an AIDS-defining illness. A less conservative medical eligibility threshold, such as that of the United States Department of Health and Human Services, which recommends that ART be started at a CD4 count of 350 cells/μl, would dramatically increase the number of eligible patients and intensify the need for rationing [5]. Even with the more conservative eligibility threshold now being applied, however, the figures in Table 1 indicate that demand for treatment will exceed supply. In the remainder of this paper, we will focus our attention on the nonmedical bases for rationing.

## Explicit Rationing Systems

In many cases, governments will set explicit criteria for which types of patients should be eligible for ART first or at lowest cost. The criteria can target selected subpopulations directly, or they can set eligibility requirements that intentionally give some patients better access than others. Possible subpopulations for direct targeting of treatment include:

### Mothers of new infants

Rather than face an ever-increasing burden of orphan support, many countries are making ART preferentially available to HIV-positive mothers through testing and treatment at antenatal clinics. The “MTCT-Plus” initiative (“MTCT” is mother-to-child transmission, and “plus” refers to an essential HIV care package for the mother in addition to strategies to reduce MTCT), which has been implemented in many African countries, is the main example of this strategy [6].

### Skilled workers

African countries face the loss of vast numbers of educated or trained workers, whose skills are vital to maintaining social welfare, sustaining output, and generating economic growth. Human capital can be conserved by giving treatment priority to nurses, teachers, engineers, judges, police officers, and other skilled workers whose contributions are important to economic development or social stability. Botswana [7], Zambia [8], and Uganda [9] have recently announced plans to target soldiers, university faculty and students, and civil servants, respectively.

### Poor people

The social justice agenda pursued by some governments and many nongovernmental organizations argues that the poorest members of society, who are least likely to be able to afford private medical care, should have preferential access to publicly funded treatment programs [10]. Means-testing, which can be applied at the level of the household or the community and calibrated to achieve the desired number of patients, is a common way to ration social benefits [11].

### High-risk populations

The extent to which ART can curb HIV transmission is a subject of current debate in the literature [12,13]. If treatment reduces the probability of transmission by suppressing viral load, then a public health argument can be made for giving preferential access to high-risk populations, such as commercial sex workers, truck drivers, or intravenous drug users.

Governments can also intentionally create eligibility requirements that result in rationing, without specifying particular target populations. Rationing systems of this type include:

### Residents of designated geographic areas

One obvious way to limit access to treatment is to offer it only to those who reside in specified geographic catchment areas [14]. These areas can be distributed around the country, centered in regions of high HIV prevalence, or concentrated in urban centers or politically important regions. Excluding patients who do not live within the designated areas may not be feasible, but most patients will not be able to afford the cost of regular transport or permanent relocation.

### Ability to co-pay

If patients are required to contribute even a small share of the cost of treatment, the number who can access therapy is likely to fall dramatically. Governments could in principle match supply and demand by setting and adjusting the level of co-payment required. The obvious outcome is a rationing system that favors the upper socioeconomic tiers of patients, who likely include the majority of skilled workers. In some societies men will also have preferential access when a cash payment is required [15]. A drawback of requiring co-payment is that poorer patients may stop therapy because they run out of funds. This is the reason for stopping cited by nearly half of all non-adherent patients in a recent study in Botswana [16].

### Commitment to adherence to therapy

Adherence to treatment regimens has been found to be the most important determinant of the success of ART at the individual patient level [17]. One way to improve the success of a large-scale treatment program, while at the same time limiting access, could therefore be to restrict therapy to patients who are judged to have the ability and willingness to adhere or who demonstrate high adherence after initiating therapy. Results of pilot projects suggest that requiring attendance at pre-treatment counseling sessions helps to screen for adherence commitment, for example [18].

## Implicit Rationing Systems

The alternative to specifying explicitly who will have priority access to resources is to allow implicit rationing systems to arise. These can be thought of as the default conditions that will prevail in the absence of explicit choices.

### Access to HIV testing

Voluntary counseling and HIV testing (VCT) is typically the entry point into an HIV/AIDS treatment program. If some subpopulations, such as youth or particular occupational groups, are targeted for HIV education and VCT services or promotion campaigns, they will have an advantage over others in seeking treatment, as will those who simply live closer to VCT facilities [19].

### Patient costs

Most countries will scale up their treatment programs incrementally, at first offering services at only a few facilities before gradually adding more. Ghana started with four public treatment sites in 2004, for example, but is aiming to have 16 in operation by the end of 2006 [20]. For most patients, bus or taxi fare will be required for regular trips to the clinic, and each trip will take up a good deal of time. Previous research has found that indirect costs due to travel time and transport play an important role in limiting access to medical care [21–24]. Unless transport is subsidized, limiting the number of service sites will effectively ration treatment to those who live nearby and to better-off households that have the resources to travel.

### First come, first served

In the absence of any other requirements, most facilities are likely to treat everyone who is medically eligible, until the supply of drugs, diagnostics, or expertise runs out. Patients who arrive after that happens may be put on a waiting list, sent to another facility, or simply sent away. This approach, which reflects an absolute shortage of treatment “slots,” is likely to favor three groups of patients: those who are already paying privately for antiretroviral drugs and shift over to publicly funded treatment once it is available; those who develop AIDS-related symptoms first, in most cases because they were infected earliest; and the few HIV-positive individuals who do not yet have AIDS but have taken the initiative to go for a test and know their own status.

### Queuing

One of the most common ways to ration scarce resources is the time-honored, time-consuming tradition of queuing. While it is possible to create a waiting list that keeps track of individuals' places in line, in many African countries the queue is a literal line outside the clinic door. Such queuing will favor patients whose opportunity cost of time is low [23]. This group is likely to be dominated by unemployed men and by women who can bring their small children with them. It may penalize employed persons and farming households that face a high seasonal demand for labor.

No matter what system is used, informal and/or illicit arrangements can often be made that give preferential access to treatment to those with social, economic, or political influence. In all of the implicit systems, and in some of the explicit ones, there will very often be a high degree of queue jumping. Elites capture a disproportionate share of resources in all countries; in developing countries, where enforcement of rules tends to be weak and informal arrangements common, it is safe to assume that members of the elite who are medically eligible for therapy will find a way to get it. De facto rationing on the basis of social or economic position will thus occur. It is the phenomenon of queue jumping that turns what appear to be equitable, if inefficient, rationing systems, such as first-come, first-served, into an inequitable and inefficient approach.

Many other potential criteria for rationing ART have been proposed or are in use [11,14,25,26]. Treatment access could be targeted, for example, to young people (because they respond best to the therapy and have their most productive years ahead of them); families of current patients (to promote adherence); those with debts (so that the loan default rate does not increase); patients with tuberculosis (to suppress transmission of tuberculosis); or children (who are least able to protect themselves).

## Evaluating the Systems

The different approaches to rationing ART described above will inevitably have very different social and economic consequences for African populations. In this section, we assess the rationing systems' probable outcomes using criteria that capture most of the principles that governments use to evaluate policies and social investments. They are by no means the sole criteria of interest, nor should they necessarily be given equal weight. We propose them only as a starting point for thinking about the consequences of alternative approaches.

### Effectiveness

Does the rationing system produce a high rate of successfully treated patients? “Successful treatment” could be defined as a fully suppressed viral load or high CD4 count over a sustained period of time. It might also incorporate some measure of viral resistance to the drugs. We assume that early diagnosis and high adherence improve effectiveness and that patient motivation improves adherence, but level of education and socioeconomic status do not [27].

### Cost savings

Is the cost per patient treated low, compared to other approaches? Cost is characterized in this way to maintain internal consistency: a rating of “high” in this domain is desirable, as it is for all other criteria. Cost is defined broadly, to incorporate costs incurred by patients, providers, insurers, and the public health system, including the identification of medically eligible patients and management of opportunistic infections, side effects, and treatment failure.

### Feasibility

Are the human and infrastructural resources needed for implementation available? We define an approach as feasible if there are no obstacles to carrying it out that appear to be insurmountable under typical conditions in sub-Saharan Africa.

### Economic efficiency

To what extent does the system mitigate the long-term impacts of the HIV epidemic on economic development? AIDS has the potential to affect economic development in many ways [28]. We focus on human capital accumulation, where human capital is defined as the accumulated skill, knowledge, and expertise of workers [29].

### Social equity

Do all medically eligible patients, including those from poor or disadvantaged subpopulations, have equal access to treatment? We define “equity” as equitable access for all at the current time, not redistribution of resources to redress past injustices, and we assume that a system's susceptibility to queue-jumping reduces its equity.

### Rationing potential

Will the chosen system sufficiently reduce the number of patients? The purpose of any rationing system is to match demand to the available supply.

### Impact on HIV transmission

To what extent does treatment reduce HIV incidence? Preferentially treating those who are likely to transmit the virus could reduce HIV incidence more than treating those who are not likely transmitters [13,30].

### Sustainability

Can the system be sustained over time? This criterion pertains to the durability of the source of funding. We assume that donor support will hold out for some time but will ultimately ebb, leaving national governments responsible for an increasing share of the costs of treatment [31].

### Effect on the health care system

How does the system for allocating ART affect the country's health care system as a whole? The choice of rationing strategies could influence whether expanding treatment access will strengthen general health services for poor communities or drain resources from non-HIV health care to meet the demand for ART, further crippling general health services.

To demonstrate how one might evaluate alternative rationing strategies, Table 2 uses the first six of these criteria to compare each of the rationing systems described above. The table omits the impact on HIV transmission and the impact on resistance, because there is little agreement on how the treatment of different populations is likely to affect these outcomes. It also omits the two long-term “systems” criteria—sustainability and effect on the health care system—because most treatment programs are so new that even informed speculation is difficult to offer.

**Table 2:**
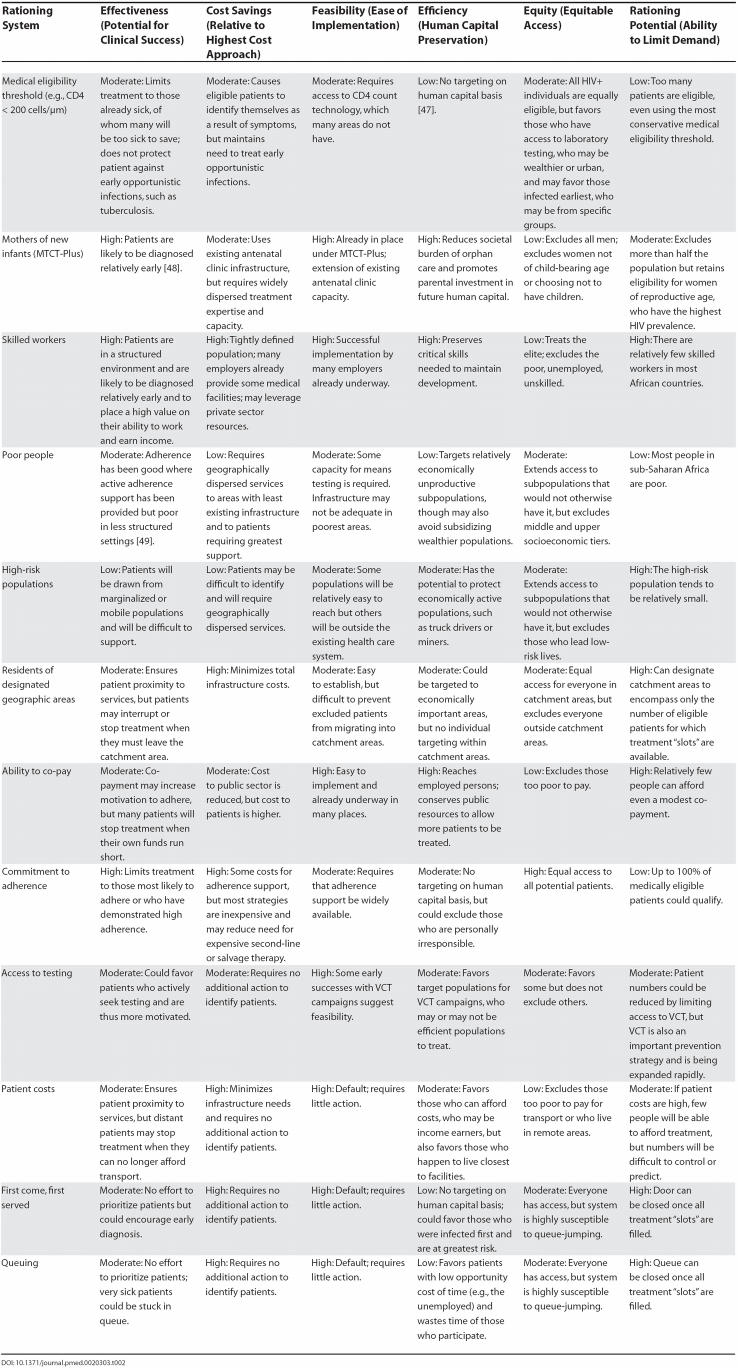
Comparison of Rationing Systems

There are several limitations to the analysis presented in Table 2. First, we do not “know” the outcomes of the strategies described above, because most of them have either not yet been tried or, if tried, have not been evaluated. Prior experience in delivering health care in sub-Saharan Africa suggests that some of our assumptions and ratings are correct. We are confident, for example, that an implicit rationing system based on queuing will result in queue-jumping, and therefore be inequitable. We are also confident that targeting skilled workers will improve labor productivity and therefore promote economic efficiency. Our ratings of some of the other systems, in contrast, are largely speculative. Table 2 also cannot capture the possibility that outcomes will vary by country, setting, or context.

A second limitation involves the criteria we selected for evaluation. We applied six criteria that we believe capture the key considerations in designing an HIV/AIDS treatment program, and we identified but did not apply three others that could alter the overall outcomes. Undoubtedly, there are other criteria that also matter. We also could not account for potential interactions among the criteria. Cost and feasibility are clearly related, for example; at some level of cost, any system could be considered feasible. Many would argue that social equity is essential to sustainable economic development, and that efficiency and equity cannot therefore be separated. While we recognize that these relationships exist, neither data nor experience allows us to address them here.

## Conclusions

During the initial months of existing ART programs in sub-Saharan Africa, limited access to health care services and widespread reluctance to be tested for HIV or enroll in treatment programs have greatly limited patient numbers [32,33]. This phenomenon, for as long as it persists, may prevent demand from exceeding supply, and no rationing will be necessary. There are already some situations, however, in which patients demanding access to care have overwhelmed available resources [34]. Even under the most optimistic scenarios for reaching universal coverage, there will be a period of at least several years when treatment is scarce.

Rationing of medical care is not a new phenomenon, nor is it by any means limited to developing countries. Waiting lists, whether for specific procedures, organs for transplant, or experimental treatments, are common in North America and Europe. Many state governments in the US are explicitly limiting access to more expensive AIDS drugs [35]. The HIV/AIDS crisis in Africa is simply bringing the need for rationing into stark relief.

There is no single rationing system, or combination of systems, that will be optimal for all countries at all times. Table 2 highlights the trade-off between economic efficiency and social equity: rationing systems that rate high in terms of efficiency generally rate low in terms of equity. African societies will place different weights on the values inherent in goals such as equity and efficiency, and decisions about rationing will be made at multiple levels of the health care system. International funding agencies have already begun to express their priorities through the amounts and conditions of their grants. Ministries of health will set policies that reflect national priorities, followed by district and local departments of health. Even individual health care workers, such as nurses at clinics where antiretroviral drugs are available but scarce, will be forced to ration access to patients who meet the clinic's or their own criteria [36].

Because access to antiretroviral drugs is a matter of life or death for patients with AIDS, the choice of rationing systems matters deeply. African governments can take one of two courses: ration deliberately, on the basis of explicit criteria, or allow implicit rationing to prevail. Implicit rationing is not likely to maximize social welfare, nor does it allow for transparency and accountability in policy making. We believe that the magnitude of the intervention now underway and the importance of the resource allocation decisions to be made call for public participation, policy analysis, and political debate in the countries affected. Several proposals have been made for how such processes could be carried out ([11,26,38,39]; A. Acharya, unpublished data). In the absence of such processes, decisions about access to treatment will be made arbitrarily and will, most likely, result in inequity and inefficiency—the worst of both worlds. Governments that make deliberate choices, in contrast, are more likely to achieve a socially desirable return from the large investments now being made than are those that allow queuing and queue-jumping to dominate. Countries that promote an open policy debate have the opportunity to ration ART in a manner that sustains both economic development and social cohesion—in the age of AIDS, the best of both worlds.

## Abbreviations

ART: antiretroviral therapy
USAID: the US Agency for International Development
VCT: voluntary counseling and HIV testing
